# The relationship between water intake and foetal growth and preterm delivery in a prospective cohort study

**DOI:** 10.1186/1471-2393-10-48

**Published:** 2010-08-24

**Authors:** J Michael Wright, Caroline S Hoffman, David A Savitz

**Affiliations:** 1 National Center for Environmental Assessment, US Environmental Protection Agency, Cincinnati, Ohio, USA; 2 Division of Extramural Research and Training, National Institute of Environmental Health Sciences, Research Triangle Park, North Carolina, USA; 3 Department of Community and Preventive Medicine, Mount Sinai School of Medicine, New York, New York, USA

## Abstract

**Background:**

Interpretation of previous associations between water intake and adverse birth outcomes is challenging given that amount and type of water consumed can be non-specific markers of exposure or underlying behavioural characteristics. We examined the relationship between water intake measures and adverse birth outcomes in participants from three study sites in the United States.

**Methods:**

Using a prospective cohort study, we examined daily intake of bottled, cold tap, total tap, and total water in relation to birth weight and risk of small-for-gestational-age (SGA) among term births and risk of preterm delivery.

**Results:**

Based on water consumption data collected between 20-24 weeks of gestation, the adjusted mean birth weight was 27 (95% confidence interval [CI]: -34, 87), 39 (95% CI: -22, 99), and 50 (95% CI: -11, 110) grams higher for the upper three total water intake quartiles (> 51-78, > 78-114, and > 114 ounces/day) compared to the lowest quartile (≤ 51 ounces/day). Adjusted birth weight results were similar for bottled water, cold tap water, and total tap water intake. An exposure-response gradient was not detected for either preterm delivery or SGA with increasing total water intake and total tap water intake, but adjusted relative risks for all three upper quartiles were below 1.0 (range: 0.6-0.9) for SGA.

**Conclusion:**

These data suggest that high water intake may be associated with higher mean birth weight following adjustment for confounding.

## Background

Water consumption is critical for metabolism, temperature regulation, transporting nutrients and wastes, and tissue maintenance. Water intake is also important for pregnant women with oligohydramnios and those at risk of developing uteroplacental insufficiency [1]. Few epidemiological studies have addressed the role of water intake on adverse reproductive outcomes with most of these focusing on the effect of specific contaminants such as disinfection by-products. Savitz *et al *[2] reported an inverse association between increased water intake and risk of preterm delivery (PTD) (ie, < 37 gestational weeks) and low birth weight infants. Compared to those reporting no daily water intake, odds ratios (ORs) were 0.5 and 0.6 for > 4 glasses/day for small for gestational age (SGA) and PTD, respectively. Relative to low intake (1-7 glasses/week), Aggazzotti *et al *[3] showed little evidence of an association between high intake of tap water (> 35 glasses/week) and risk of SGA or PTD (ORs = 1.0 and 1.1, respectively). Other studies have shown a decreased risk of spontaneous abortion [4] and cardiac anomalies [5] with increased bottled water intake. Given that water intake is a non-specific marker of exposure, it is not clear if these results are due to residual confounding or actual effects of water ingestion.

Using a prospective cohort study, we examined birth weight and risk of SGA among term births and risk of PTD in relation to daily bottled, cold tap, total tap and total water intake. The primary study hypothesis examined whether water intake is associated with measures of foetal growth and protective of adverse birth outcomes.

## Methods

### Study design and population

The study population included 2766 pregnant women enrolled in a prospective cohort study conducted from December 2000-May 2004 across three study sites in the United States. Participants were enrolled early in pregnancy (≤ 12 weeks' gestation) or while planning to become pregnant. Eligible subjects included those who were ≥18 years of age, who did not have any fertility treatment for the study pregnancy, and who intended to deliver in the study area. Additional details on study design and recruitment have been published elsewhere [6,7]. The Institutional Review Boards at the University of North Carolina, University of Tennessee and the University of Texas approved the study protocols; participants gave informed consent.

The sample size for the PTD analysis was 2039 pregnancies after the following exclusions: 259 with a missing or incomplete baseline interview, 347 with a pregnancy loss, 90 that were lost to follow-up, 16 with repeat live births, eight with multiple births, and seven with missing information on date of birth or birth weight. 1854 live births were available for the birth weight analysis, and 1783 live term births were available for the SGA analysis due to missing information on maternal race/ethnicity, births with a reported maternal race of "Indian," "Asian/Pacific Islander," or "Other," and births delivered at < 25 or > 42 weeks' gestation.

### Assessment of foetal growth and PTD

Infant date of birth, birth weight, and gender were obtained from medical records for 43% of live births, from vital records for 57%, and from participant self-report for < 1%. Self-reported last menstrual period (LMP) and an early ultrasound (scheduled between gestational weeks 6-7 and no later than 14 weeks), both obtained during the first trimester, were combined with infant date of birth to estimate gestational age at birth. Gestational age derived from LMP was used for the majority of subjects (81%) unless the LMP date was incomplete (1%) or differed by more than ±7 days from the ultrasound-based estimate of gestational age (18%), in which case the ultrasound estimate was used. SGA was defined as an infant with a birth weight below the tenth percentile for gestational age at birth, gender, maternal race/ethnicity (non-Hispanic white, non-Hispanic black, or Hispanic), and parity based on United States population estimates [8,9].

### Assessment of exposures and confounding factors

Data on exposures and potential confounding factors were collected via telephone interviews before 16 weeks of gestation (baseline interview) and between 20-24 weeks of gestation (follow-up interview). The interviews included detailed questions about the pregnancy, maternal health, demographic information, behavioural characteristics, and water use practices. Maternal health characteristics included pre-pregnancy body mass index (BMI) defined as weight/height^2 ^and categorized according to the Institute of Medicine's [10] guidelines: low (< 19.8 kg/m^2^), normal (19.8-26.0 kg/m^2^), overweight (26.1-29.0 kg/m^2^), and obese (> 29.0 kg/m^2^). Behavioural variables included recreational exercise, smoking, intake of caffeine, vitamins, alcohol, and illicit drugs. Caffeine intake from beverages (ie, coffee, tea, and soda) was estimated and then categorized using the cut points 150 mg/day and 300 mg/day [11].

At each interview, study participants were asked how many bottles of water and glasses/cups of cold tap water, hot tap water and tap water-based drinks (including juice, coffee, tea, and other beverages they made from tap water) they consumed each day during a typical week. Participants were asked to define their glass or cup sizes according to three options: small (0.1-0.3 L), medium (0.4-0.6 L), or large (0.7-1.0 L) for cold tap and bottled water and small (0.1-0.3 L), medium (0.3-0.5 L), or large (0.5-0.7 L) for hot water. The midpoint for each size range was used to estimate water consumption in ounces/day. Bottled water included spring water, mineral water, distilled water, sparkling water or any water purchased in bottles or plastic jugs or obtained from a water cooler. Bottled water intake was calculated as the average amount based on reported container sizes: small (8-12 ounces), medium (14-24 ounces), and large (26-34 ounces). Among the women working outside the study area (8%), the tap water ingestion question was asked separately for consumption at home and at work. This resulted in a higher average total amount for this group. We, therefore, deflated their cold tap water consumption totals by 15.3% and hot tap water consumption by 18.2% for those reporting work and home totals separately to make their mean values equal to those women who reported the aggregated amount.

Follow-up data and an average of the baseline and follow-up data were used to examine the following exposure measures: cold tap water intake, total tap water (cold and hot) intake, bottled water intake, and total water (tap and bottled) intake. Water use measures were divided into quartiles and analyzed using the lowest quartile as the referent. Based on self-reported data collected during the follow-up questionnaire, women were classified into the following quartiles for total water intake: 0-51 (referent), > 51-78, > 78-114, and > 114 ounces/day. Women were classified into the following quartiles for total tap water intake: 0-30 (referent), > 30-61, > 61-96, and > 96 ounces/day. Due to limited data on bottled water intake in the population, this variable was dichotomized to allow comparison of any versus no bottled water intake.

### Statistical analysis

We calculated risk ratios (RRs) and 95% confidence intervals (CIs) for SGA and preterm delivery based on various water consumption measures using Poisson regression with robust error variance. The association with term birth weight was examined using linear regression. We considered potential confounding variables that were associated with the outcomes and were independently associated with water use exposures, but were not intermediates in the causal pathway between exposure and disease: maternal age, race/ethnicity, education level, annual household income, employment status, marital status, pre-pregnancy BMI, parity, alcohol consumption, smoking status, caffeine intake, vitamin intake, recreational activity, swimming, infant gender, season of birth, and study site. Maternal education, race/ethnicity, income, infant gender, parity, pre-pregnancy BMI, smoking, vitamin intake, employment during pregnancy, and study site were retained in multivariate models as confounders using a change-in-estimate (10% change) backwards elimination approach. We examined the extent that confounding impacted the birth weight results among those variables retained in the backwards elimination model. Relative to the unadjusted models for total water intake from the follow-up data, we examined percent change-in-estimate for the linear regression models for the confounders that were retained in multivariate models.

## Results

The mean birth weight in the total population (n = 2039) was 3382 grams, with 9% (n = 192) born preterm (Table 1). Among the 1854 term births, 5% (n = 85) were classified as SGA. Lower mean birth weights were found among infants born to mothers who were non-Hispanic black, of younger age, were not married, had lower BMI, were less educated, and had an annual household income < $30,000. Large differences in mean birth weight were also detected between Site 1 versus Site 3 (165 grams), non-smokers versus smokers (325 grams), and vitamin users versus non-users (159 grams). Several factors were associated with both higher mean birth weight and higher water intake. For example, mothers of non-Hispanic white ethnicity, higher BMI, and those who consumed vitamins tended to have larger infants and drink larger amounts of total water, so that adjustment for these factors attenuated the association between water intake and birth weight. Using a change-in estimate analysis, we examined the individual contribution of various confounders in the birth weight model for total water intake based on the follow-up data. Compared to the univariate results, the largest average change-in estimates across the highest three exposure quartiles were swimming (39%), maternal race/ethnicity (31%), annual household income (21%), education (20%), marital status (20%), and vitamin intake (17%).

**Table 1:** Characteristics of the study population recruited from three US cities during 2000-4

Population characteristics			PTD	SGA^a^	Birth weight (g)	
			
	n	%	%	%	Mean	SD
Total population	2039	100	9	5	3382	586
Maternal race/ethnicity						
Non-Hispanic white	1169	57	7	5	3486	596
Non-Hispanic black	609	30	12	7	3167	545
Hispanic	185	9	9	6	3427	632
Other	73	4	7	--	3400	459
Missing	3					
Maternal age (years)						
< 25	599	29	11	7	3255	558
25-29	657	32	8	6	3393	572
30-34	564	28	7	4	3486	587
≥35	219	11	11	6	3425	642
Highest maternal education level						
High school or less	573	28	13	9	3235	616
Some college	440	22	10	5	3340	600
College degree or higher	1025	50	7	4	3482	544
Maternal smoking						
Yes	99	5	16	12	3073	578
No	1940	95	9	5	3398	583
Maternal alcohol use						
Yes	32	2	9	6	3410	702
No	2007	98	6	6	3381	585
Pre-pregnancy BMI (kg/m^2^)						
< 19.8	232	11	8	9	3291	535
19.8-25.9	1016	50	8	5	3410	553
26.0-29.9	333	16	7	6	3421	535
> 29.9	407	20	14	5	3349	713
Missing	51	3	10	16	3255	771
Vitamin use						
Yes	1027	50	7	5	3461	573
No	1012	50	11	7	3302	590
Caffeine intake (mg/day)						
None	519	25	9	4	3410	613
1-150	468	23	8	5	3385	557
151-300	387	19	11	6	3344	617
> 300	665	33	9	7	3380	567
Marital status						
Married	1390	68	7	5	3466	536
Not married	648	32	13	8	3200	646
Missing	1					
Parity						
Nulliparous	991	49	10	6	3323	591
Parous	1048	51	8	4	3438	577
Employed during past 4 months						
Yes	1430	70	9	5	3372	580
No	608	30	9	7	3406	602
Annual household income ($)						
< 30,000	637	31	12	7	3245	629
30,001-60,000	535	26	7	7	3437	545
60,001-80,000	321	16	6	5	3460	498
> 80,000	465	23	8	3	3504	571
Missing	81	4	20	10	3078	614
Recreational activity^b^						
Yes	1109	54	9	6	3423	572
No	930	46	10	5	3332	600
Vigorous recreational activity^c^						
Yes	401	20	8	7	3412	559
No	1638	80	9	5	3374	593
Swimming						
Yes	651	58	6	6	3471	549
No	1388	32	10	6	3340	599
Infant gender						
Male	1045	51	9	7	3419	585
Female	994	49	9	5	3343	586
Study site						
Site 1	929	46	6	5	3459	581
Site 2	761	37	11	6	3329	593
Site 3	349	17	13	8	3294	562

^a^Term births only

^b^Any recreational physical activity or exercise, such as brisk walking, jogging, swimming, biking, tennis, soccer, or dancing

^c^Any recreational physical activity or exercise, such as brisk walking, jogging, swimming, biking, tennis, soccer, or dancing that caused large increases in breathing and heart rate

SGA = small for gestational age; PTD = preterm delivery; SD = standard deviation; BMI = body mass index

### Birth weight

Compared to the lowest quartile, the unadjusted mean birth weight in grams was higher for the upper three quartiles of total water intake, 60 (95% CI: -1, 122), 67 (95% CI: 5, 128), and 83 (95% CI: 21, 145) grams, respectively (Table 2). Following adjustment for confounding (by study site, household income, maternal education, maternal race/ethnicity, infant gender, parity, pre-pregnancy BMI, smoking, vitamin use, and employment during pregnancy), the respective adjusted differences in mean birth weight in grams were reduced to 27 (95% CI: -34, 87), 39 (95% CI: -22, 99), and 50 (95% CI: -11, 111) grams compared to the lowest quartile. When total water intake was examined as a continuous measure (per 20 ounce/day increased intake), the adjusted increase in mean birth weight was 7.3 (95% CI: -0.8, 15.5) grams. Adjusted results were similar in magnitude for the upper quartiles of cold tap water intake, total tap water intake, and total water intake for both the follow-up data and an average of the follow-up and baseline data. Compared to no bottled water intake, the adjusted mean birth weight for bottled water consumers was 31 grams (95% CI: -20, 82) based on follow-up data and 43 grams (95% CI: -27, 113) based on an average of the follow-up and baseline data.

**Table 2:** Birth weight results for daily bottled, cold tap, total tap, and total water intake

	Follow-up data					Average of baseline and follow-up data	
	
		Unadjusted		**Adjusted**^a^		**Adjusted**^a^	
		
Exposure	n (%)	β	95% CI	β	95% CI	β	95% CI
Bottled water							
None	448 (25%)	Ref		Ref		Ref	
Any	1329 (75%)	12	(-38, 63)	31	(-20, 82)	43	(-27, 113)
Cold tap water (*ounces*)^b^							
0-27	424 (24%)	Ref		Ref		Ref	
> 27-53	455 (25%)	12	(-50, 74)	9	(-53, 72)	25	(-38, 88)
> 53-91	444 (25%)	74	(11, 136)	52	(-11, 116)	44	(-19, 107)
> 91	453 (26%)	77	(14, 139)	49	(-14, 111)	65	(2, 128)
Per 20 ounce		12.3	(3.9, 20.7)	8.5	(0.1, 16.9)	8.5	(-1.5, 18.5)
Total tap water (*ounces*)^b^							
0-30	443 (25%)	Ref		Ref		Ref	
> 30-61	445 (25%)	41	(-21, 103)	44	(-18, 106)	10	(-52, 73)
> 61-96	496 (28%)	100	(40, 161)	78	(17, 139)	34	(-30, 97)
> 96	392 (22%)	85	(21, 149)	43	(-21, 107)	46	(-17, 109)
Per 20 ounce		12.0	(4.0, 20.1)	6.8	(-1.3, 15.0)	5.3	(-4.3, 15.1)
Total water (*ounces*)^b^							
0-51	452 (25%)	Ref		Ref		Ref	
> 51-78	439 (25%)	60	(-1, 122)	27	(-34, 87)	10	(-50, 71)
> 78-114	442 (25%)	67	(5, 128)	39	(-22, 99)	55	(-6, 116)
> 114	441 (25%)	83	(21, 145)	50	(-11, 111)	37	(-25, 98)
Per 20 ounce		9.8	(1.6, 18.1)	7.3	(-0.8, 15.5)	5.2	(-4.7, 15.2)

^a^Adjusted for race, education level, annual household income, smoking, pre-pregnancy BMI, vitamin use, parity, employed during last 4 months, infant gender, and study site

^b^Categorical exposure cutpoints for average data results were slightly higher than the follow-up data

BMI= body mass index.

### SGA

RRs and 95% CIs comparing women who reported drinking > 51-78, > 78-114, and > 114 versus 0-51 ounces of total water per day at follow-up were 0.7 (0.4, 1.2), 0.6 (0.3, 1.0), and 0.8 (0.5, 1.4), indicative of a decreased risk of SGA with increased water consumption above the first quartile but no gradient thereafter (Table 3). Results were similar following adjustment for confounding: 0.8 (0.4, 1.4), 0.6 (0.3, 1.0), and 0.9 (0.5, 1.6), respectively. Relative to the lowest quartile, adjusted RRs were slightly higher for cold tap and total tap water intake especially for the average follow-up and baseline data. Relative to women not drinking bottled water, the adjusted RR for SGA was 0.9 (95% CI: 0.5, 1.4) based on the follow-up data and 1.4 (0.6, 3.0) based on the average follow-up and baseline data.

**Table 3:** Small-for-gestational-age results^a ^for daily bottled, cold tap, total tap, and total water intake

	Follow-up data			Average of baseline and follow-up data
**Exposure**	**n (%)**	**Unadjusted RR (95% CI)**	**Adjusted RR (95% CI)**^b^	**Adjusted RR (95% CI)**^b^

Bottled water				
None	448 (26%)	1	1	1
Any	1258 (74%)	1.1 (0.7, 1.7)	0.9 (0.5, 1.4)	1.4 (0.6, 3.0)
Cold tap water (*ounces*)^c^				
0-27	403 (24%)	1	1	1
> 27-53	439 (26%)	0.9 (0.5, 1.5)	1.2 (0.6, 2.3)	1.1 (0.6, 2.2)
> 53-91	429 (25%)	0.9 (0.5, 1.6)	1.3 (0.7, 2.4)	1.4 (0.7, 2.6)
> 91	439 (26%)	0.7 (0.4, 1.2)	0.9 (0.5, 1.9)	1.1 (0.5, 2.1)
Per 20 ounce		1.0 (0.9, 1.0)	1.0 (0.9, 1.1)	1.0 (0.9, 1.1)
Total tap water (*ounces*)^c^				
0-30	423 (25%)	1	1	1
> 30-61	426 (25%)	0.7 (0.4, 1.3)	0.9 (0.5, 1.7)	1.2 (0.6, 2.2)
> 61-96	476 (28%)	0.7 (0.4, 1.2)	0.8 (0.5, 1.6)	1.3 (0.7, 2.6)
> 96	380 (22%)	0.7 (0.4, 1.2)	0.9 (0.5, 1.9)	1.1 (0.6, 2.2)
Per 20 ounce		1.0 (0.9, 1.1)	1.0 (0.9, 1.1)	1.0 (0.9, 1.1)
Total water (*ounces)*^c^				
0-51	434 (25%)	1	1	1
> 51-78	418 (25%)	0.7 (0.4, 1.2)	0.8 (0.4, 1.4)	0.9 (0.5, 1.6)
> 78-114	424 (25%)	0.6 (0.3, 1.0)	0.6 (0.3, 1.0)	0.5 (0.2, 1.0)
> 114	427 (25%)	0.8 (0.5, 1.4)	0.9 (0.5, 1.6)	1.0 (0.6, 1.8)
Per 20 ounce		1.0 (0.9, 1.1)	1.0 (0.9, 1.1)	1.0 (0.9, 1.1)

^a^Term SGA models restricted to infants born to non-Hispanic White, non-Hispanic Black, or Hispanic women

^b^Adjusted for race, education level, annual household income, smoking, pre-pregnancy BMI, vitamin use, parity, employed during last 4 months, infant gender, and study site

^c^Categorical exposure cutpoints for average data results were slightly higher than the follow-up data

BMI= body mass index; RR= risk ratio

### PTD

As shown in Table 4, RRs and 95% CIs for PTD comparing women who reported drinking > 51-78, > 78-114, and > 114 versus 0-51 ounces of total water per day based on the follow-up data were 1.0 (0.7, 1.6), 1.0 (0.7, 1.6), and 1.2 (0.8, 1.8). RRs were slightly larger following adjustment for confounding: 1.2 (0.7, 1.9), 1.1 (0.7, 1.8), and 1.4 (0.9, 2.2), respectively. RRs and 95% CIs for PTD for the cold tap and total tap water quartiles were generally below 1.0 compared to the lowest quartile for follow-up data and average follow-up and baseline data. Relative to women not drinking bottled water, the adjusted RR for any bottled water intake was 1.2 (95% CI: 0.8, 1.8) for the follow-up data and 0.8 (95% CI: 0.5, 1.4) based on the average baseline and follow-up data.

**Table 4:** Preterm delivery results for daily bottled, cold tap, total tap, and total water intake

	Follow-up data			Average of baseline and follow-up data
**Exposure**	**n (%)**	**Unadjusted RR (95% CI)**	**Adjusted RR (95% CI)**^a^	**Adjusted RR (95% CI)**^a^

Bottled water				
None	483 (25%)	1	1	1
Any	1458 (75%)	1.2 (0.9, 1.8)	1.2 (0.8, 1.8)	0.8 (0.5, 1.4)
Cold tap water (*ounces*)^b^				
0-27	472 (24%)	1	1	1
> 27-53	494 (25%)	0.8 (0.5, 1.2)	0.8 (0.5, 1.3)	0.9 (0.6, 1.4)
> 53-91	485 (25%)	0.9 (0.6, 1.3)	0.9 (0.6, 1.4)	0.9 (0.6, 1.4)
> 91	490 (25%)	0.8 (0.5, 1.1)	0.8 (0.5, 1.3)	1.0 (0.6, 1.5)
Per 20 ounce		1.0 (0.9, 1.0)	0.9 (0.7, 1.3)	0.9 (0.6, 1.3)
Total tap water (*ounces*)^b^				
0-30	493 (25%)	1	1	1
> 30-61	483 (25%)	0.8 (0.5, 1.2)	0.8 (0.5, 1.2)	1.0 (0.6, 1.5)
> 61-96	535 (28%)	0.7 (0.5, 1.1)	0.8 (0.5, 1.2)	1.2 (0.8, 1.9)
> 96	429 (22%)	0.9 (0.6, 1.3)	1.0 (0.6, 1.5)	0.9 (0.6, 1.4)
Per 20 ounce		1.0 (0.9, 1.0)	0.9 (0.7, 1.2)	0.9 (0.6, 1.3)
Total water (*ounces*)^b^				
0-51	491 (25%)	1.0	1.0	1.0
> 51-78	478 (25%)	1.0 (0.7, 1.6)	1.2 (0.7, 1.9)	1.1 (0.7, 1.7)
> 78-114	481 (25%)	1.0 (0.7, 1.6)	1.1 (0.7, 1.8)	1.0 (0.6, 1.5)
> 114	478 (25%)	1.2 (0.8, 1.8)	1.4 (0.9, 2.2)	1.2 (0.8, 1.9)
Per 20 ounce		1.0 (1.0, 1.1)	1.1 (0.9, 1.4)	1.1 (0.8, 1.5)

^a^Adjusted for race, education level, annual household income, smoking, pre-pregnancy BMI, vitamin use, parity, employed during last 4 months, infant gender, and study site

^b^Categorical exposure cutpoints for average data results were slightly higher than the follow-up data

BMI= body mass index; RR= risk ratio

## Discussion

Previous epidemiological studies of disinfection by-products in drinking water have shown a possible increased risk of impaired foetal growth with increasing exposure but a decreased risk of PTD [12-16]. Hoffman *et al *[13] postulated that the decreased risk of PTD may be partially due to a protective effect of higher water intake on pregnancy outcomes, but this has rarely been examined. Overall, the adjusted SGA results in our study were largely null although the RRs for follow-up data on total tap water and total water intake were consistently below 1.0 (range 0.6-0.9) for the higher water exposure categories. The adjusted PTD findings were also largely null with most RRs below the null value of 1.0. For both the follow-up and average of follow-up and baseline data analyses, we observed a slight increased risk of PTD in the high total water intake group (RR's = 1.2 and 1.4) relative to those in the low intake category.

To the best of our knowledge, this is the first study to examine the relationship between water intake and birth weight in a prospective epidemiological study. We saw some evidence of an exposure-response relationship with mean birth weight differences ranging from 27-50 grams with increasing total water intake compared to the lowest quartile after adjustment. We found results similar in magnitude for the cold tap water and total tap water intake measures based on the follow-up data. Similar results were found for all three exposure measures based on an average of baseline and follow-up questionnaire data including suggestion of an exposure-response relationship for cold tap and total tap water intake.

One of the strengths of the study was the detailed individual-level information on water intake and potential confounding factors collected for this pregnancy cohort. Several of these confounders had considerable impact on the association between water intake and birth weight. For example, established risk factors for foetal growth measures such as maternal race, age, education, and household income attenuated the mean birth weight by 17-39% compared to the univariate total water intake model results. Although we did not find evidence that diabetes was a strong confounder in this analysis, results were slightly stronger when the birth weight data were restricted to non-diabetics (data not shown). We recognize that even after adjustment for confounding factors, there may be unmeasured aspects of maternal physiology or behaviour that affect both water intake and pregnancy outcome so that the water intake itself is not causally related to the outcomes. For example, previous research suggests that participants reporting no water intake also reported increased soft drink consumption and less fruit, vegetable and low- and medium-fat dairy product intake [17]. Although dietary information was not collected on this population, our detailed analysis of confounding likely led to indirect control of nutritional status to some degree through adjustment of confounders such as household income, education, and prenatal vitamin use. In addition, very few study participants (0.3%) in our study population reported no water intake which should minimize the potential for confounding due to unhealthy lifestyles during pregnancy.

An additional study strength was the collection of multiple measures of water use during pregnancy which allowed for examination of water intake measures. This is potentially important for exposure assessment as previous studies have indicated that water use changes may occur during pregnancy [18]. It is not entirely clear, however, whether these changes are due to behavioural decisions related to perceived health benefits, physiologic changes such as increased thirst, or variation due to measurement error. Since 95% of foetal growth occurs after the 20th gestational week, [19] we considered the follow-up data (collected during 20-24 gestational weeks) to be the most relevant data for examination of foetal growth measures and prematurity. However, we also examined water intake results based on a measure of the average follow-up/baseline data, but saw little difference in comparison to the follow-up data.

Although these data represent one of the most extensive water use data collective efforts to date in a reproductive epidemiological study, self-reported data are subject to recall error. Given the prospective nature of the data collection, differential error could not have occurred since the birth outcomes were unknown at the time the pregnant subjects reported the water consumption and information on confounding factors. Nonetheless, water intake is difficult to measure and may be subject to non-differential error. This may have reduced our statistical power and limited the ability to detect exposure-response relationships and effects small in magnitude (e.g., small changes in mean birth weight). Another limitation of the study was a narrow exposure gradient for bottled water intake which precluded examination of multiple exposure categories. In contrast, there was considerable variability in reported tap and total water intake across study subjects, but we did not have a truly unexposed (ie, those not consuming any water) reference group for the total tap and total water categories. The number of subjects (0.3%) reporting no water intake in this population is less than that from other studies (5-12%) [2,17,20].

Our study subjects were highly motivated, highly educated, and represented a low risk population, since they were actively seeking prenatal care during pregnancy and volunteered for this study. This may limit the generalizability of study findings and also raises the potential for bias among this highly motivated population if drinking water or other "healthful behaviours" are related to self-selection. The overall proportion of preterm births in the study population is lower than (9% vs. 13%) that reported in the United States in 2005 [21]. The prevalence of SGA (5%) in our population based on birth weight deciles from United States population estimates was also lower than would be expected for the general population. This low risk population, therefore, limited our statistical power to detect associations due to the decreased frequency of adverse health outcomes (e.g., SGA) being considered. SGA may also include small births that are both pathologically growth restricted and some that are constitutionally small due to a variety of factors such as maternal ethnicity, parity, weight, height, etc. Therefore, the examination of SGA births is a potential limitation that could limit our ability to detect associations that may be present if some of these births represent constitutionally small births that are not truly growth restricted.

## Conclusions

In conclusion, we found limited evidence of an association between specific measures of water intake and risk of adverse pregnancy outcomes such as SGA and PTD. This is in contrast to a previous study which reported an inverse association with water intake and risk of both SGA and PTD, [2] but is consistent with another study which found no association between water intake and risk of either PTD or low birth weight [3]. Despite limited statistical power, we did see some evidence of small increases in mean birth weight for higher levels of total water intake during pregnancy. This might warrant further examination in higher risk populations as this was the first study to examine this endpoint in relation to water intake.

## Competing interests

The authors declare that they have no competing interests.

## Authors' contributions

The design of cohort study was contributed by DAS. The scope and purpose of the paper was developed by all of the authors. The analyses were conducted by JMW and CSH. The text was written and reviewed by all of the authors.

## Pre-publication history

The pre-publication history for this paper can be accessed here:

http://www.biomedcentral.com/1471-2393/10/48/prepub
